# Feasibility of state of the art PET/CT systems performance harmonisation

**DOI:** 10.1007/s00259-018-3977-4

**Published:** 2018-03-02

**Authors:** Andres Kaalep, Terez Sera, Sjoerd Rijnsdorp, Maqsood Yaqub, Anne Talsma, Martin A. Lodge, Ronald Boellaard

**Affiliations:** 1 Department of Medical Technology, North Estonia Medical Centre Foundation, J. Sutiste Str 19, 13419 Tallinn, Estonia; 2 Department of Nuclear Medicine, University of Szeged, Szeged, Hungary; 3 On behalf of EANM Research Limited (EARL), Vienna, Austria; 4 Department of Medical Physics, Catharina Hospital, Eindhoven, The Netherlands; 5 Department of Radiology and Nuclear Medicine, VU University Medical Center, Amsterdam, The Netherlands; 6 Department of Radiology, Martini Hospital, Groningen, Netherlands; 7 Russell H. Morgan Department of Radiology and Radiological Science, Johns Hopkins University, Baltimore, MD 21287 USA; 8 Department of Nuclear Medicine and Molecular Imaging, University of Groningen, University Medical Centre Groningen, Hanzeplein 1, Groningen, the Netherlands

**Keywords:** Performance, Harmonisation, PET/CT, Quantification, EARL accreditation

## Abstract

**Purpose:**

The objective of this study was to explore the feasibility of harmonising performance for PET/CT systems equipped with time-of-flight (ToF) and resolution modelling/point spread function (PSF) technologies. A second aim was producing a working prototype of new harmonising criteria with higher contrast recoveries than current EARL standards using various SUV metrics.

**Methods:**

Four PET/CT systems with both ToF and PSF capabilities from three major vendors were used to acquire and reconstruct images of the NEMA NU2–2007 body phantom filled conforming EANM EARL guidelines. A total of 15 reconstruction parameter sets of varying pixel size, post filtering and reconstruction type, with three different acquisition durations were used to compare the quantitative performance of the systems. A target range for recovery curves was established such that it would accommodate the highest matching recoveries from all investigated systems. These updated criteria were validated on 18 additional scanners from 16 sites in order to demonstrate the scanners’ ability to meet the new target range.

**Results:**

Each of the four systems was found to be capable of producing harmonising reconstructions with similar recovery curves. The five reconstruction parameter sets producing harmonising results significantly increased SUVmean (25%) and SUVmax (26%) contrast recoveries compared with current EARL specifications. Additional prospective validation performed on 18 scanners from 16 EARL accredited sites demonstrated the feasibility of updated harmonising specifications. SUVpeak was found to significantly reduce the variability in quantitative results while producing lower recoveries in smaller (≤17 mm diameter) sphere sizes.

**Conclusions:**

Harmonising PET/CT systems with ToF and PSF technologies from different vendors was found to be feasible. The harmonisation of such systems would require an update to the current multicentre accreditation program EARL in order to accommodate higher recoveries. SUVpeak should be further investigated as a noise resistant alternative quantitative metric to SUVmax.

**Electronic supplementary material:**

The online version of this article (10.1007/s00259-018-3977-4) contains supplementary material, which is available to authorized users.

## Introduction

^18^F–fluorodeoxyglucose (18F–FDG) positron emission tomography (PET) and computed tomography (CT) hybrid imaging (PET/CT) is an important functional imaging tool being widely used for diagnosis, staging and therapy response evaluation in, e.g., oncology [1–20]. Combined anatomical and functional information can be obtained in one session using hybrid PET/CT. In clinical practice, visual inspection of PET/CT images might be sufficient for the purposes of staging or restaging [7, 21], however PET is a quantitative technique [22–26] and can provide more accurate and less observer-dependent metrics for diagnosis, therapy assessment and response monitoring using quantitative data in addition to visual interpretation [27]. In recent oncological clinical trials quantitative PET/CT data are also used for patient selection, stratification and therapy response monitoring. However, variability, reproducibility and accuracy of quantitative PET/CT imaging [28–34] have to be considered. Scientific societies such as the European Association of Nuclear Medicine (EANM), American College of Radiology (ACR), American Association of Physicists in Medicine (AAPM), Radiological Society of North America (RSNA) and Society of Nuclear Medicine and Molecular Imaging (SNMMI) are closely collaborating to promote standardisation of practices in order to reduce variability of quantification in multicentre clinical trials. Initiatives such as QIBA-UPICT, SNMMI-CTN and EANM-EARL are providing quality control programs to assure quantitative comparability [35–40].

High utilisation of PET/CT in oncology can be attributed to the availability of 18F–FDG [5, 41]. Dynamic PET scans and pharmacokinetic modelling to evaluate the rate of glucose metabolism of tumours is an excellent method for quantification [27] but the technical impediments such as the limited scanner field of view and increased scan acquisition time make it unfeasible for routine use [42]. In clinical practice, a simplified uptake metric such as the standard uptake value (SUV) [43, 44] is therefore most commonly used. While SUV analysis is relatively easy to apply, it suffers from multiple technical, physical and biological factors that can significantly affect quantification [27]. The required level of harmonisation depends on the intended use of the PET study. When the same PET/CT system is used for therapy assessment and based on relative changes in SUV before and after therapy, a high reproducibility rather than absolute accuracy might be most important. It has been shown that in this case, when the scanner performance remains unchanged over time, consistent application of a certain methodology could be sufficient [34, 45]. However, patients are often scanned on different PET/CT systems, either because the scanner had been replaced by a new one, or in different institutions, which makes accurate cross-calibration of systems a crucial requirement. Absolute quantitative measures (e.g., residual uptake of 18F–FDG after therapy session) are also being used for differentiation between malignant and benign lesions, determining prognosis and response monitoring [27]. This again requires high reproducibility and comparability of the quantitative data, especially in multicentre settings.

One of the challenges in PET/CT systems performance harmonisation is the variability caused by different PET/CT technologies available in the field. Multicentre standards should not be based on the less performing systems; they need to fit with the highest, yet common denominator in systems’ performance. Additionally, in case of optimization of PET/CT systems performance for lesion detection, a single centre quantification does not necessarily coincide with a multicentre one. A particular challenge for recent PET/CT systems resulted from the introduction of time-of-flight (ToF) and resolution modelling (point spread function (PSF)) capabilities. The latter increased tumour detectability but also caused higher variability across centres, since some have and others lack these technologies. Currently a large number of the EARL accredited PET/CT systems [46] do not have PSF image reconstruction capabilities. However, it is expected that over the next couple of years the majority of the PET/CT systems will be equipped with these new reconstruction techniques.

The aim of this paper is to explore the feasibility of harmonising performance of PET/CT systems equipped with the latest PET technologies such as TOF and PSF, which were recently commercially released.

## Materials and methods

### PET/CT system selection

Four PET/CT systems equipped with both ToF and PSF capabilities from three major vendors (General Electric (GE), Siemens and Philips) were selected for this study. Systems included were the Siemens Biograph mCT (Siemens system 1), the Siemens Biograph mCT Flow (Siemens system 2), the GE Discovery 710 (GE system) and the Philips Ingenuity TF 128 (Philips system). The equipment was calibrated in accordance with the corresponding manufacturer’s instructions. In addition, all systems were participating and accredited in the EANM/EARL 18F–FDG PET/CT accreditation program. Detailed specifications for the systems can be found in supplemental Table 1 and references [47–51].

### Phantom experiments

The phantoms and filling procedures used complied with the EANM/EARL guidelines for Image Quality QC measurements which need to be performed annually as part of the EANM/EARL accreditation program [35]. The NEMA NU2–2007 body phantom was used, which is a plastic cylinder in the form of a fillable torso cavity, to act as a background compartment. It has a 5 cm diameter cylindrical lung insert in the centre and six fillable spheres with internal diameters of 10, 13, 17, 22, 28 and 37 mm, positioned coaxially around the lung insert. The lung insert is filled with polystyrene beads in order to mimic lung tissue. The phantom background compartment and the spherical inserts were filled with 18F–FDG solutions aimed at activity concentrations of 2 kBq/mL and 20 kBq/mL, respectively, at the start of the measurements, resulting in a sphere to background activity concentration ratio of 10:1.

### Acquisition and reconstruction parameters

In accordance with current EANM/EARL guidelines for 18F–FDG Image Quality QC phantom imaging [35], a low dose CT acquisition, followed by an emission scan consisting of two bed positions with an acquisition time of 5 min per bed position is to be acquired for the “image quality” dataset to assess contrast recovery performance. In this study, acquisition time of 5 min per bed position was selected as the reference for high count statistics. In order to investigate the effect of reduced count statistics on contrast recovery, data acquired with shorter acquisition times, respectively 2 and 1 min per bed position, were collected. The GE and Philips systems had list mode data acquisition capability available, which meant that only the 5 min/bed position emission scans were acquired and reconstructions with shorter acquisition times were generated retrospectively from the list mode data. On the Siemens systems included in this study, multiple shorter emission scans were acquired with the phantom left in an unchanged position. In order to facilitate the Siemens Flow system’s (Siemens system 2) possibility of performing scanning with continuous table movement, instead of a specific bed position scanning duration, table feed speeds of 0.5 mm/s, 1 mm/s and 2 mm/s were selected, resulting in similar acquisition times as with the other scanners.

Reconstructions were performed using the software available on each of the PET/CT systems. TOF, PSF, normalisation, randoms, scatter and attenuation corrections were applied and the reconstruction parameters were selected to increase overall contrast recovery, meanwhile aiming at achieving comparable recovery values across systems (for each sphere). In addition, we also considered achieving comparable recovery values between the spheres to minimise severe partial volume effects as well as large Gibbs overshoots. Clinically used and vendor recommended reconstruction parameters were applied and varied. Three iterations with 21 subsets were used for Siemens 1 (Biograph mCT) and two iterations with 21 subsets for Siemens 2 (mCT Flow) reconstruction. For GE - B, D, F and G (Discovery 710) - two iterations with 24 subsets and the VPFXS reconstruction method were used, while for GE - A, C and E - the QCFX reconstruction method, with an unknown number of iterations and subsets, was used. For the Philips systems the iterations/subsets were 3/33 but these could not be selected prior to scanning, with no values retrieved from the DICOM header of the images; so the BLOB OS TF reconstruction method was used. Different Gaussian filters and pixel sizes within clinically relevant ranges were also investigated in order to study their effects on contrast recovery. Additionally, for the GE system, a proprietary reconstruction method, the “Q.Clear”, which uses a Bayesian penalised-likelihood reconstruction algorithm, was investigated using different penalization factors (β) and its effect on quantitative image quality was evaluated. Due to differences among vendors and models, the available reconstruction parameters and their ranges were limited based on availability and/or user selectability. In total, 15 reconstruction parameter sets (reconstruction modes) were used to assess and compare the quantitative performance of the investigated systems. Each reconstruction mode was applied on three different scans, acquired with long (~4 min/bed for the Siemens Flow system; ~5 min/bed for all other systems), with medium (~2 min/bed) and short (~1 min/bed) frame durations. A summary of the acquisition and reconstruction settings of the 15 reconstruction modes is presented in Table 1.

**Table 1:** Acquisition and reconstruction settings for the initial 15 reconstruction modes

Reconstruction mode	Post filter width (mm)	Q.Clear β value	Pixel size (mm)	Slice thickness (mm)	Long frame duration (s)	Medium frame duration (s)	Short frame duration (s)
GE - A	N/A	200	2.73	3.27	300	120	60
GE - B	0	N/A	2.73	3.27	300	120	60
GE - C	N/A	350	2.73	3.27	300	120	60
GE - D	3	N/A	2.73	3.27	300	120	60
GE - E	N/A	800	2.73	3.27	300	120	60
GE - F	5	N/A	2.73	3.27	300	120	60
GE - G	6.4	N/A	2.73	3.27	300	120	60
Philips - A	N/A	N/A	2.00	2.00	301	120	60
Philips - B	N/A	N/A	4.00	4.00	301	120	60
Siemens 1 - A	0	N/A	2.04	2.00	300	120	60
Siemens 1 - B	0	N/A	1.59	2.00	300	120	60
Siemens 1 - C	3	N/A	2.04	2.00	300	120	60
Siemens 1 - D	5	N/A	2.04	2.00	300	120	60
Siemens 1 - E	6.5	N/A	3.18	2.00	300	120	60
Siemens 2 - A	5	N/A	4.07	5.00	223	111	56

### Data analysis

Data reconstructed on the PET/CT were exported to a PC for further analysis using the EARL semi-automatic tool [35] designed for quantitative analysis of images of the NEMA NU2–2007 body phantom, filled conforming to EANM/EARL guidelines for 18F–FDG Image Quality QC phantom imaging. The software tool requires phantom images in DICOM format and filling data as input, and extracts SUV recovery for the spheres, a calibration factor for the background compartment and standard deviation and coefficients of variation from uniform images of the background. The SUV recovery coefficient (RC) is defined as the ratio between measured and expected activity concentration in each spherical insert. RC values were calculated based on 50% background corrected isocontour VOI (RC_SUVmean_), maximum voxel value included in VOI (RC_SUVmax_) and spherical VOI with a diameter of 12 mm, positioned so to yield the highest uptake (RC_SUVpeak_) [35, 39, 52].

Prior to further analysis, all data were corrected for system calibration bias in order to be able to compare the various reconstruction modes’ impact on RCs and not to be effected by inter-scanner calibration errors. For this purpose, to all RCs a correction factor, defined as the ratio between expected and measured activity concentration in the corresponding uniform background compartment, was applied. For the 15 initial reconstruction modes, inter-scanner global correction factors ranged from 0.88 to 1.12, with the mean and standard deviation being 0.98 and 0.055, respectively. Intra-scanner changes were below 1%. For the 23 additional reconstructions, the inter-scanner global correction factors ranged from 0.93 to 1.10 (one system, however, showed a correction factor of 0.8), with the mean and standard deviation values of 0.99 and 0.055, respectively.

### Selection of harmonising reconstruction modes

The primary objective of this study was to find reconstruction modes providing high, yet uniform contrast recoveries within the spheres of the NEMA NU2–2007 body phantom, which could be matched across all generations of PET/CT systems currently used in clinical practice – which would result in quantitative harmonisation of PET/CT systems.

**Fig. 1:**
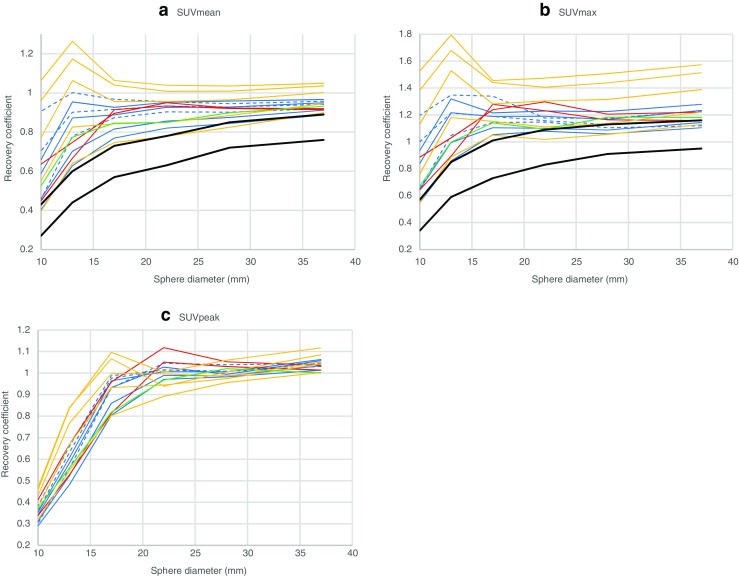
RC curves derived from 15 initial reconstruction modes using SUVmean (**a**), SUVmax (**b**) and SUVpeak (**c**) quantitative metrics. Only long acquisition time frame curves are displayed. GE (Q.Clear) – blue dashed lines, GE (non-Q.Clear) – blue solid lines, Philips – red solid lines, Siemens 1 – orange solid lines, Siemens 2 – green solid lines, current EARL specifications – black solid lines

RC_SUVmean_, RC_SUVmax_ and RC_SUVpeak_ curves for all reconstructed phantom images were plotted against sphere diameters (Fig. 1) and characterised using visual and quantitative analysis, for which the applied metrics are summarised in Table 2. Reconstruction modes with higher RCs than current EARL specifications, as well as tightly grouped and stable RC_SUVmean_ and RC_SUVmax_ curves, were sought for harmonisation purposes.

**Table 2:** Description of quantitative metrics used

Metric	Description of metric
SUV_mean_	Ratio of image derived average radioactivity concentration within a region of interest and the whole body concentration of the injected radioactivity
SUV_max_	Ratio of image derived maximum (single pixel) radioactivity concentration within a region of interest and the whole body concentration of the injected radioactivity
SUV_peak_	Ratio of image derived average radioactivity concentration within a 12 mm diameter spherical volume within the region of interest, positioned to yield the highest uptake, and the whole body concentration of the injected radioactivity
RC	Recovery Coefficient - the ratio between image derived and expected activity concentration
MCR*	Mean Contrast Recovery - mean RC of all spheres in corresponding reconstruction mode’s long duration acquisition. Parameter is indicative of reconstruction mode’s overall contrast recovery potential.
CoV_MCR_	Coefficient of Variation (SD/mean*100, %) of a group of MCR values. Parameter is indicative of RC curves’ alignment within a group.
CRV_medium_*	Contrast Recovery Variability - Mean deviation of medium duration acquisition spheres’ RCs from the corresponding values of long duration aquisition.
CRV_short_*	Contrast Recovery Variability - Mean deviation of short duration acquisition spheres’ RCs from the corresponding values of long duration aquisition.
CoV_BG_*	Coefficient of Variation (SD/mean *100, %) of measured activity concentration within the uniform background compartment of the phantom. Parameter is indicative of the noise present in the images.
Curvature	Long acquisition duration root-mean-square deviation of spheres’ RC values from RC value of the largest (37 mm) sphere. Parameter characterises the deviation of smaller spheres’ RC values which usually cause the RC-object size relation to assume a curved shape.
Absolute error	Long acquisition duration root-mean-square deviation of spheres’ RC values from unity. The parameter characterises the reconstruction mode’s ability to report accurate activity concentration values.
Curvature (excl. 10 mm sphere)	Same as "curvature" but excluding the smallest (10 mm) sphere.
Absolute error (excl. 10 mm sphere)	Same as "absolute error" but excluding the smallest (10 mm) sphere.

*Quantitative metrics that were retrospectively used to determine harmonising cut-off criteria

The harmonising reconstruction modes were selected by simultaneously analysing quantitative characteristics of the reconstruction modes along with visual appearance of the RC curves. The following considerations were kept in mind while determining feasible reconstruction modes – (1) the proposed harmonising specifications should provide an increase over the current EARL compliant RC values, (2) the bandwidth of RCs should be similar to the current Earl specification limits and (3) the harmonising RC curves should not demonstrate major overshoots (=upward bias) due to Gibbs artefacts. While the harmonising reconstruction modes were selected based on the abovementioned considerations, quantitative cut-off criteria were retrospectively determined and stated in Table 9 based on the bandwidth and characteristics of harmonising reconstruction modes. Performances of the candidate reconstruction modes were compared with the initial group of reconstructions as well as current EARL accreditation specifications.

#### Mean contrast recovery (MCR)

Mean contrast recovery (MCR) was calculated in order to evaluate overall contrast recovery potential of a reconstruction mode while Coefficient of Variation of the MCR parameter (CoV_MCR_) was used to characterise agreement among various reconstruction modes’ RC curves. Increased coinciding MCR and reduced CoV_MCR_ values were preferred.

#### Contrast recovery variability (CRV)

Contrast Recovery Variability (CRV_medium_ and CRV_short_) parameters were used to evaluate a reconstruction mode’s ability to produce consistent results in case of reduced count statistics. In order to achieve it, RCs of short and medium time frame acquisitions were compared to the long acquisition’s corresponding spheres’ RCs and relative differences calculated. Lower values were deemed preferable as being indicative of reconstruction mode’s stability and reduced variability in noisy environments.

#### Noise

Image noise was quantitatively evaluated by measuring the Coefficient of Variation (%, SD/Mean*100) in the uniform background compartment (CoV_BG_) for each reconstruction mode and acquisition time frame. CoV_BG_ cut-off limit of 15%, based on the existing EARL guideline and UPICT [35, 37, 40], was implemented to determine suitable reconstruction modes for harmonisation. Reconstruction modes providing lower noise images were deemed preferable.

#### Curvature and absolute error

Curvature and absolute error parameters were used to evaluate RC variability and absolute accuracy of RC measurements due to changes in sphere/lesion size. Reduced values were preferable, but similar magnitude across systems/reconstructions was given priority.

#### Visual analysis

Visual analysis of the RC curves was used to identify reconstruction modes that exhibited abnormal behaviour or localised variations, such as exaggerated Gibbs artefacts, that were not identified by the previously described quantitative parameters.

The reconstruction modes, which were considered for harmonisation based on SUVmean and SUVmax performance, were also used to develop provisional specifications for SUVpeak.

### Validation of reconstruction modes for harmonisation

In order to prospectively evaluate the reproducibility and inter-scanner variability of the proposed reconstruction modes for harmonisation, 16 EARL accredited facilities, equipped with current generation PET/CT systems, participated in the study and provided the requested reconstructions from independent phantom acquisitions applying acquisition and reconstruction parameters (supplemental Table 2) identical or similar to the reconstructions proposed for harmonisation purposes. Data received from the centres was analysed in the same way as the reconstructions in the pilot study.

## Results

### New specifications proposed for harmonisation

**Fig. 2:**
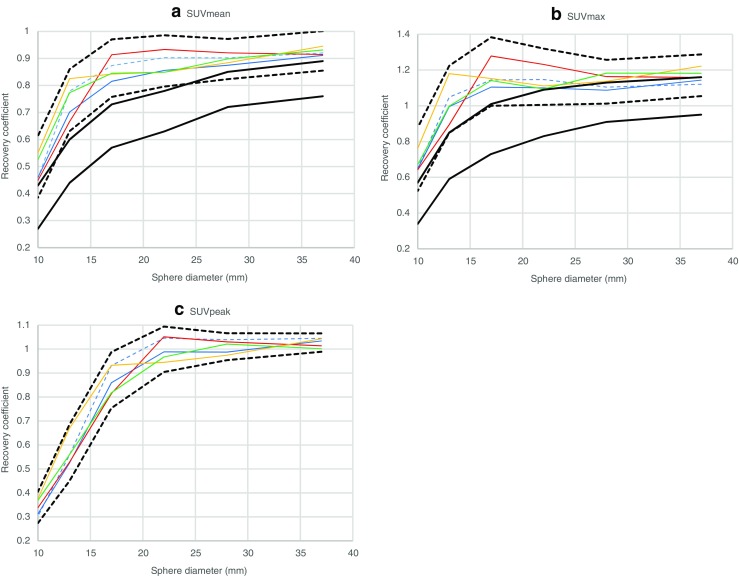
RC curves derived from suggested harmonising reconstruction modes using SUVmean (**a**), SUVmax (**b**) and SUVpeak (**c**) quantitative metrics along with current EARL and possible new specifications. Only long acquisition time frame curves are displayed. GE (Q.Clear) – blue dashed lines, GE (non-Q.Clear) – blue solid lines, Philips – red solid lines, Siemens 1 – orange solid lines, Siemens 2 – green solid lines, current EARL specifications – black solid lines, possible new EARL specifications – black dashed lines

Analysis of the initial 15 reconstruction modes resulted in five reconstruction modes, which produced the highest uniform contrast recoveries and were feasible for all of the investigated systems considering SUVmean and SUVmax (Philips - B, GE – E, GE - F, Siemens 1 – D and Siemens 2 – A), to be considered for harmonisation. In order to accommodate unavoidable inter-scanner variability and reproducibility errors due to equipment calibration and user inaccuracy, all of the RC ranges were expanded to be proportional (i.e., using the same bandwidth of performance, but taking into account increased contrast recovery) to current EARL specifications for sphere recoveries. Bandwidths for proposed and current EARL specifications as well as the RC curves derived from the five reconstruction modes are presented in Fig. 2. For the provisional SUVpeak specifications, average sphere recoveries of the five reconstruction modes and a bandwidth of ±2 standard deviations was used. Additionally, recovery coefficients are plotted as a function of background noise for each sphere and per SUVmetric (presented in supplemental Figs. 4–6). Axial slices of the phantom data from the five harmonising reconstructions are shown in supplemental Fig. 7.

#### Mean contrast recovery (MCR)

SUVmean and SUVmax RC curves vary substantially among different systems and reconstruction modes as seen in Fig. 1 and Tables 3 and 4. The reconstruction mode showing the lowest recoveries (Siemens 1 – E) produced a SUVmean MCR value of 0.714 and SUVmax MCR of 0.948 while for the highest recovery reconstruction mode (Siemens 1 – A), the corresponding values were 1.09 and 1.56 – a difference of more than 50%. SUVpeak MCR values were found to be between 0.754 and 0.929. CoV_MCR_ values for the 15 reconstruction modes were 12.4% and 15.4% for SUVmean and SUVmax, respectively, while for SUVpeak, CoV_MCR_ was 6.0%.

**Table 3:**
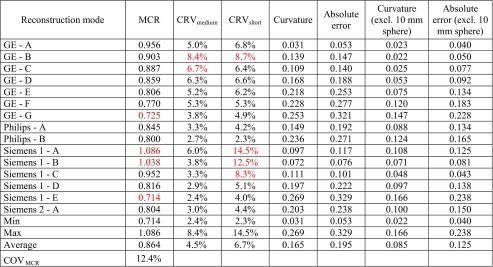
Analysis results of 15 initial reconstruction modes using a SUVmean quantitative metric. Values found to be outside of acceptable range during retrospective quantitative analysis, are coloured red

**Table 4:**
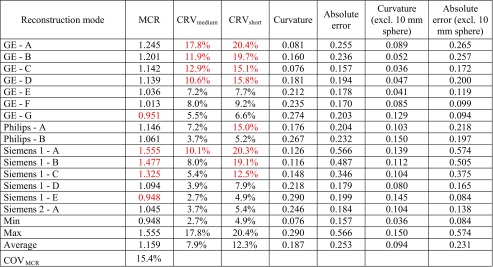
Analysis results of 15 initial reconstruction modes using a SUVmax quantitative metric. Values found to be outside of acceptable range during retrospective quantitative analysis, are coloured red

For the five reconstruction modes proposed for harmonisation, the range of MCR values were 0.770–0.816 and 1.01–1.09 for SUVmean and SUVmax, respectively. The harmonising reconstruction modes produced SUVpeak MCR values in the range of 0.784–0.823. CoV_MCR_ values for SUVmean, SUVmax and SUVpeak were 2.2%, 2.9% and 2.2%, respectively.

#### Contrast recovery variability (CRV)

The initial 15 reconstruction modes demonstrated a variable sensitivity as a function of count statistics. The expected increase in variability with decrease in count statistics was observed in all reconstruction modes by comparing CRV_medium_ and CRV_short_ values (Tables 3, 4 and 5). The CRV_medium_ results for SUVmean, SUVmax and SUVpeak ranged from 2.4% to 8.4%, 2.7% to 17.8% and 1.6% to 4.5%, respectively. The CRV_short_ results for SUVmean, SUVmax and SUVpeak ranged from 2.3% to 14.5%, 4.9% to 20.4% and 2.7% to 6.3%, respectively.

**Table 5:** Analysis results of 15 initial reconstruction modes using SUVpeak quantitative metric

Reconstruction mode	MCR	CRV_medium_	CRV_short_	Curvature	Absolute error	Curvature (excl. 10 mm sphere)	Absolute error (excl. 10 mm sphere)
GE - A	0.848	3.9%	3.7%	0.334	0.287	0.187	0.153
GE - B	0.833	3.4%	5.7%	0.381	0.310	0.237	0.179
GE - C	0.840	2.3%	3.6%	0.359	0.302	0.211	0.166
GE - D	0.823	3.9%	6.3%	0.389	0.320	0.248	0.191
GE - E	0.821	2.9%	4.1%	0.400	0.339	0.250	0.203
GE - F	0.784	3.3%	5.8%	0.404	0.346	0.272	0.223
GE - G	0.757	3.1%	5.9%	0.413	0.367	0.287	0.248
Philips - A	0.874	3.2%	3.4%	0.328	0.281	0.192	0.161
Philips - B	0.796	2.8%	2.9%	0.383	0.341	0.263	0.229
Siemens 1 - A	0.901	4.5%	6.3%	0.305	0.232	0.148	0.090
Siemens 1 - B	0.929	1.6%	4.2%	0.325	0.240	0.154	0.103
Siemens 1 - C	0.872	3.3%	5.0%	0.308	0.251	0.151	0.107
Siemens 1 - D	0.823	3.0%	4.5%	0.350	0.291	0.204	0.155
Siemens 1 - E	0.754	3.9%	2.7%	0.382	0.346	0.255	0.226
Siemens 2 - A	0.789	2.9%	4.9%	0.355	0.323	0.240	0.214
Min	0.754	1.6%	2.7%	0.305	0.232	0.148	0.090
Max	0.929	4.5%	6.3%	0.413	0.367	0.287	0.248
Average	0.830	3.2%	4.6%	0.361	0.305	0.220	0.177
COV_MCR_	6.0%						

For the five reconstruction modes proposed for harmonisation, the CRV_medium_ results for SUVmean, SUVmax and SUVpeak ranged from 2.7% to 5.3%, 3.7% to 8.0% and 2.8% to 3.0%, respectively. The CRV_short_ results for SUVmean, SUVmax and SUVpeak ranged from 2.3% to 6.2%, 5.2% to 9.2% and 2.9% to 5.8%, respectively (Tables 6, 7 and 8).

**Table 6:** Results of the analysis of five reconstruction modes considered for harmonisation using the SUVmean quantitative metric

Reconstruction mode	MCR	CRV_medium_	CRV_short_	Curvature	Absolute error	Curvature (excl. 10 mm sphere)	Absolute error (excl. 10 mm sphere)
GE - E	0.806	5.2%	6.2%	0.218	0.253	0.075	0.134
GE - F	0.770	5.3%	5.3%	0.228	0.277	0.120	0.183
Philips - B	0.800	2.7%	2.3%	0.236	0.271	0.124	0.165
Siemens 1 - D	0.816	2.9%	5.1%	0.197	0.222	0.097	0.138
Siemens 2 - A	0.804	3.0%	4.4%	0.203	0.238	0.100	0.150
Min	0.770	2.7%	2.3%	0.197	0.222	0.075	0.134
Max	0,816	5.3%	6.2%	0.236	0.277	0.124	0.183
Average	0.799	3.8%	4.6%	0.216	0.252	0.103	0.154
COV_MCR_	2.2%						
EARL min	0.570	N/A	N/A	0.282	0.466	0.198	0.393
EARL max	0.710	N/A	N/A	0.277	0.342	0.176	0.251
EARL Average	0.640	N/A	N/A	0.279	0.403	0.187	0.321

**Table 7:** Results of the analysis of five reconstruction modes considered for harmonisation using the SUVmax quantitative metric

Reconstruction mode	MCR	CRV_medium_	CRV_short_	Curvature	Absolute error	Curvature (excl. 10 mm sphere)	Absolute error (excl. 10 mm sphere)
GE - E	1.036	7.2%	7.7%	0.212	0.178	0.041	0.119
GE - F	1.013	8.0%	9.2%	0.235	0.170	0.085	0.099
Philips - B	1.061	3.7%	5.2%	0.267	0.232	0.150	0.197
Siemens 1 - D	1.094	3.9%	7.9%	0.218	0.179	0.080	0.165
Siemens 2 - A	1.045	3.7%	5.4%	0.246	0.184	0.104	0.138
Min	1.013	3.7%	5.2%	0.212	0.170	0.041	0.099
Max	1.094	8.0%	9.2%	0.267	0.232	0.150	0.197
Average	1.050	5.3%	7.1%	0.236	0.189	0.092	0.144
COV_MCR_	2.9%						
EARL min	0.730	N/A	N/A	0.347	0.355	0.220	0.237
EARL max	0.970	N/A	N/A	0.339	0.236	0.176	0.121
EARL Average	0.850	N/A	N/A	0.342	0.277	0.198	0.142

**Table 8:** Results of the analysis of five reconstruction modes considered for harmonisation using the SUVpeak quantitative metric

Reconstruction mode	MCR	CRV_medium_	CRV_short_	Curvature	Absolute error	Curvature (excl. 10 mm sphere)	Absolute error (excl. 10 mm sphere)
GE - E	0.821	2.9%	4.1%	0.400	0.339	0.250	0.203
GE - F	0.784	3.3%	5.8%	0.404	0.346	0.272	0.223
Philips - B	0.796	2.8%	2.9%	0.383	0.341	0.263	0.229
Siemens 1 - D	0.823	3.0%	4.5%	0.350	0.291	0.204	0.155
Siemens 2 - A	0.789	2.9%	4.9%	0.355	0.323	0.240	0.214
Min	0.784	2.8%	2.9%	0.350	0.291	0.204	0.155
Max	0.823	3.3%	5.8%	0.404	0.346	0.272	0.229
Average	0.803	3.0%	4.4%	0.378	0.328	0.246	0.205
COV_MCR_	2.2%						

#### Noise

The CoV_BG_ values are summarised in supplemental Fig. 8. The average CoV_BG_ of all reconstruction modes with a long time frame was 12.6%. For medium and short acquisition times, the corresponding values were 19.7% and 27.0%, respectively. The selected reconstruction modes for harmonisation purposes produced average CoV_BG_ values of 9.4%, 14.0% and 18.4% for long, medium and short acquisition time frames, respectively.

#### Curvature and absolute error

Curvatures for the initial 15 reconstruction modes were in the ranges of 0.031–0.269, 0.076–0.290 and 0.305–0.413 for SUVmean, SUVmax and SUVpeak, respectively. For the five reconstruction modes suggested for harmonisation, the SUVmean, SUVmax and SUVpeak curvatures were in the ranges of 0.197–0.236, 0.212–0.267 and 0.350–0.404, respectively.

Absolute errors for the initial 15 reconstruction modes were in the ranges of 0.053–0.329, 0.157–0.566 and 0.232–0.367 for SUVmean, SUVmax and SUVpeak, respectively. For the five reconstruction modes selected for harmonisation, the SUVmean, SUVmax and SUVpeak curvatures ranged between 0.222–0.277, 0.170–0.232 and 0.291–0.346, respectively.

#### Visual analysis

Significant variations in investigated RC curves’ shapes and positions of Siemens 1 - A, B, C, GE - A, B, C, D and Philips – A reconstruction modes were noticed when compared with other systems or acquisition times and considered unsuitable for harmonisation. Based on the bandwidth and characteristics of harmonising reconstruction modes, quantitative cut-off criteria were determined and are stated in Table 9.

**Table 9:** Retrospectively determined quantitative cut-off criteria for the harmonising reconstructions

	SUVmean	SUVmax
MCR	±11% (0.77–0.96)	±13% (1.01–1.31)
CRV_medium_	6%	8%
CRV_short_	7%	9%
Visual analysis	No excessive Gibbs and partial volume artefacts	
Noise	Background CoV ≤15% (high statistics acquisition)	

### Additional reconstructions

**Fig. 3:**
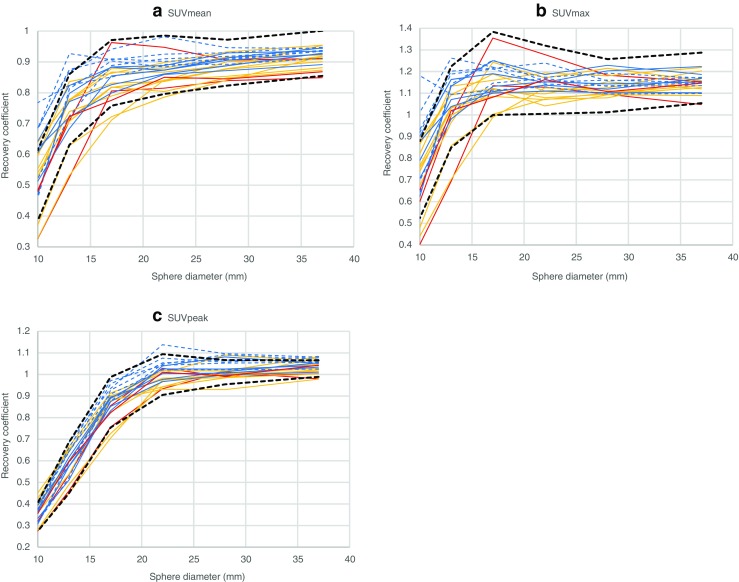
RC curves derived from additional reconstructions using SUVmean (**a**), SUVmax (**b**) and SUVpeak (**c**) quantitative metrics along with proposed new specifications. GE (Q.Clear) – blue dashed lines, GE (non-Q.Clear) – blue solid lines, Philips – red solid lines, Siemens – orange solid lines, possible new EARL specifications – black dashed lines

Sixteen EARL accredited sites participated in the prospective evaluation of the newly proposed specifications for harmonisation and performed reconstructions according to instructions provided. Data received included 23 distinctive reconstructions from three GE Discovery 710 systems, two Philips Ingenuity systems, six Siemens mCT systems, three Siemens mCT Flow systems, one GE Discovery IQ system, two GE Discovery MI systems and one Philips Vereos system. RC curves derived from the 18 systems along with proposed new harmonising specifications can be seen in Fig. 3. For SUVmean, 16 out of 138 analysed spheres produced RC values outside of the suggested accreditation interval, while for SUVmax and SUVpeak, the number of outliers was 12. Quantitative results describing additional reconstructions can be found in Tables 10, 11 and 12. Specifications, based on the current findings, proposed for harmonisation along with current EARL specifications are presented in Table 13.

**Table 10:** Analysis results of 23 additional reconstructions using the SUVmean quantitative metric

PET/CT system	MCR	CRV_medium_	CRV_short_	Curvature	Absolute error	Curvature (excl. 10 mm sphere)	Absolute error (excl. 10 mm sphere)
Ingenuity 1	0.820	N/A	N/A	0.213	0.249	0.106	0.145
Ingenuity 2	0.694	N/A	N/A	0.276	0.365	0.164	0.263
mCT Flow 1	0.691	N/A	N/A	0.303	0.368	0.196	0.270
mCT Flow 2	0.711	N/A	N/A	0.298	0.339	0.190	0.242
mCT Flow 3	0.816	N/A	N/A	0.193	0.231	0.079	0.136
mCT 1	0.847	N/A	N/A	0.176	0.194	0.080	0.112
mCT 2	0.786	N/A	N/A	0.194	0.250	0.115	0.181
mCT 3	0.825	N/A	N/A	0.188	0.208	0.113	0.142
mCT 4	0.765	N/A	N/A	0.174	0.262	0.091	0.195
mCT 5	0.786	N/A	N/A	0.195	0.245	0.119	0.179
mCT 6	0.811	N/A	N/A	0.136	0.207	0.078	0.161
Discovery 710 1	0.847	N/A	N/A	0.153	0.182	0.079	0.120
Discovery 710 2	0.793	N/A	N/A	0.217	0.254	0.129	0.174
Discovery 710 1 Q.Clear 1	0.887	N/A	N/A	0.120	0.145	0.027	0.074
Discovery 710 2 Q.Clear 2	0.817	N/A	N/A	0.211	0.236	0.110	0.146
Discovery 710 3 Q.Clear 3	0.895	N/A	N/A	0.121	0.144	0.042	0.073
GE Discovery MI 1	0.794	N/A	N/A	0.150	0.228	0.099	0.182
GE Discovery MI 2	0.813	N/A	N/A	0.171	0.214	0.102	0.155
GE Discovery MI 1 Q.Clear 1	0.857	N/A	N/A	0.081	0.151	0.055	0.129
GE Discovery MI 2 Q.Clear 2	0.869	N/A	N/A	0.118	0.156	0.039	0.096
GE Discovery IQ 1	0.817	N/A	N/A	0.219	0.244	0.077	0.123
GE Discovery IQ 1 Q.Clear 1	0.818	N/A	N/A	0.221	0.246	0.069	0.118
Vereos 1	0.757	N/A	N/A	0.191	0.277	0.087	0.195
Min	0.691			0.081	0.144	0.027	0.073
Max	0.895			0.303	0.368	0.196	0.270
Average	0.805			0.188	0.235	0.098	0.157
COV_MCR_	6.6%

**Table 11:** Analysis results of 23 additional reconstructions using the SUVmax quantitative metric

PET/CT system	MCR	CRV_medium_	CRV_short_	Curvature	Absolute error	Curvature (excl. 10 mm sphere)	Absolute error (excl. 10 mm sphere)
Ingenuity 1	1.094	N/A	N/A	0.278	0.264	0.143	0.228
Ingenuity 2	0.917	N/A	N/A	0.334	0.288	0.188	0.167
mCT Flow 1	0.911	N/A	N/A	0.347	0.270	0.207	0.159
mCT Flow 2	0.943	N/A	N/A	0.350	0.234	0.187	0.109
mCT Flow 3	1.071	N/A	N/A	0.237	0.211	0.110	0.179
mCT 1	1.118	N/A	N/A	0.185	0.179	0.057	0.179
mCT 2	1.038	N/A	N/A	0.173	0.140	0.065	0.108
mCT 3	1.098	N/A	N/A	0.168	0.148	0.082	0.151
mCT 4	1.019	N/A	N/A	0.160	0.130	0.041	0.082
mCT 5	1.033	N/A	N/A	0.176	0.127	0.067	0.092
mCT 6	1.067	N/A	N/A	0.113	0.107	0.033	0.105
Discovery 710 1	1.139	N/A	N/A	0.151	0.176	0.051	0.188
Discovery 710 2	1.045	N/A	N/A	0.213	0.168	0.086	0.130
Discovery 710 1 Q.Clear 1	1.172	N/A	N/A	0.085	0.189	0.054	0.207
Discovery 710 2 Q.Clear 2	1.049	N/A	N/A	0.204	0.172	0.064	0.131
Discovery 710 3 Q.Clear 3	1.154	N/A	N/A	0.114	0.184	0.042	0.200
GE Discovery MI 1	1.055	N/A	N/A	0.105	0.100	0.032	0.095
GE Discovery MI 2	1.066	N/A	N/A	0.179	0.142	0.065	0.125
GE Discovery MI 1 Q.Clear 1	1.119	N/A	N/A	0.040	0.123	0.017	0.108
GE Discovery MI 2 Q.Clear 2	1.124	N/A	N/A	0.107	0.157	0.039	0.168
GE Discovery IQ 1	1.102	N/A	N/A	0.255	0.240	0.047	0.201
GE Discovery IQ 1 Q.Clear 1	1.083	N/A	N/A	0.234	0.219	0.052	0.177
Vereos 1	1.029	N/A	N/A	0.230	0.176	0.074	0.115
Min	0.911			0.040	0.100	0.017	0.082
Max	1.172			0.350	0.288	0.207	0.228
Average	1.063			0.193	0.180	0.078	0.148
COV_MCR_	6.3%

**Table 12:** Analysis results of 23 additional reconstructions using SUVpeak quantitative metric

PET/CT system	MCR	CRV_medium_	CRV_short_	Curvature	Absolute error	Curvature (excl. 10 mm sphere)	Absolute error (excl. 10 mm sphere)
Ingenuity 1	0.789	N/A	N/A	0.376	0.341	0.246	0.218
Ingenuity 2	0.736	N/A	N/A	0.405	0.383	0.284	0.267
mCT Flow 1	0.737	N/A	N/A	0.439	0.390	0.324	0.280
mCT Flow 2	0.750	N/A	N/A	0.476	0.379	0.353	0.263
mCT Flow 3	0.797	N/A	N/A	0.393	0.328	0.274	0.217
mCT 1	0.858	N/A	N/A	0.348	0.282	0.214	0.162
mCT 2	0.812	N/A	N/A	0.347	0.302	0.225	0.188
mCT 3	0.847	N/A	N/A	0.365	0.281	0.242	0.169
mCT 4	0.781	N/A	N/A	0.326	0.313	0.198	0.192
mCT 5	0.803	N/A	N/A	0.355	0.304	0.243	0.199
mCT 6	0.827	N/A	N/A	0.297	0.269	0.184	0.163
Discovery 710 1	0.829	N/A	N/A	0.357	0.301	0.234	0.188
Discovery 710 2	0.794	N/A	N/A	0.398	0.342	0.274	0.227
Discovery 710 1 Q.Clear 1	0.867	N/A	N/A	0.372	0.294	0.231	0.171
Discovery 710 2 Q.Clear 2	0.824	N/A	N/A	0.413	0.344	0.276	0.221
Discovery 710 3 Q.Clear 3	0.884	N/A	N/A	0.370	0.298	0.212	0.166
GE Discovery MI 1	0.797	N/A	N/A	0.351	0.313	0.233	0.202
GE Discovery MI 2	0.819	N/A	N/A	0.375	0.308	0.237	0.180
GE Discovery MI 1 Q.Clear 1	0.838	N/A	N/A	0.328	0.285	0.200	0.166
GE Discovery MI 2 Q.Clear 2	0.859	N/A	N/A	0.356	0.294	0.202	0.157
GE Discovery IQ 1	0.814	N/A	N/A	0.407	0.342	0.263	0.210
GE Discovery IQ 1 Q.Clear 1	0.831	N/A	N/A	0.412	0.336	0.258	0.199
Vereos 1	0.803	N/A	N/A	0.381	0.320	0.251	0.199
Min	0.736			0.297	0.269	0.184	0.157
Max	0.884			0.476	0.390	0.353	0.280
Average	0.813			0.376	0.320	0.246	0.200
COV_MCR_	4.7%

**Table 13:** SUVmean, SUVmax and SUVpeak specifications proposed for harmonisation along with current EARL specifications

Sphere diameter (mm)	Current EARL RC bandwidth			Proposed RC bandwidth		
	SUVmean	SUVmax	SUVpeak	SUVmean	SUVmax	SUVpeak
37	0.76–0.89	0.95–1.16	N/A	0.85–1,00	1.05–1.29	0.99–1.07
28	0.72–0.85	0.91–1.13	N/A	0.82–0.97	1.01–1.26	0.95–1.07
22	0.63–0.78	0.83–1.09	N/A	0.80–0.99	1.01–1.32	0.90–1.09
17	0.57–0.73	0.73–1.01	N/A	0.76–0.97	1.00–1.38	0.75–0.99
13	0.44–0.60	0.59–0.85	N/A	0.63–0.86	0.85–1.22	0.45–0.69
10	0.27–0.43	0.34–0.57	N/A	0.39–0.61	0.52–0.88	0.27–0.41

## Discussion

The SUVmean and SUVmax RC curves of the initial 15 reconstruction modes vary significantly, even within one system. This reflects the high degree of variability that could be introduced into quantitative PET with variation in reconstruction settings. The selection of harmonising reconstruction modes, and the validation which followed on additional reconstructions, demonstrated that the variability can be reduced to acceptable limits.

The acquisition time of 5 min per bed position specified in the current EARL accreditation settings, while characterising system performance in high statistics scenarios, may not provide an accurate representation of the reconstruction mode’s performance in clinical settings. Therefore, the observation of reduced CRV_medium_ and CRV_short_ in reconstruction modes for harmonisation is important since the acquisition times when utilising new PET/CT systems are routinely reduced to 2 min or less per bed position.

Significant increase in both SUVmean and SUVmax MCR values was observed in the reconstruction modes proposed for harmonisation compared to the corresponding current EARL specifications. The trend is in agreement with results recently published by Sunderland et al. demonstrating that high-end PET/CT systems are having significantly increased SUVmax values in anthropomorphic phantom scans [53]. The metrics for all of the spheres demonstrated a noticeable increase; however, for the smaller spheres (≤ 17 mm) the effect was relatively stronger. This could be explained by the so-called Gibbs artefact which produces an overshoot of measured activity at the edges of the spheres, becoming more dominant at smaller sizes, also described by Lasnon et al. [54]. To some extent the effect can be considered beneficial, compensating for the inherently lower recoveries seen in the smaller spheres. It should, however, be noticed that with the use of resolution modelling (PSF) without any or with minimal post filtering applied, the overshoot could introduce significant positive SUV bias, in particular when using SUVmax. Methods like regularised (MAP) reconstruction with a regularising prior (such as Q.Clear implemented by GE) can also be used to suppress Gibbs artefacts and were therefore also considered in this study.

The increased SUVmean and SUVmax recoveries seen in the proposed reconstruction modes for harmonisation would significantly reduce the gap that exists today between standardised quantitative reconstruction protocols used in multicentre settings and the locally developed non-standard protocols for lesion detection and general visual assessment – both of which are used in parallel in many nuclear medicine departments. Close agreement between the two could lead to the adoption of a single reconstruction mode that would provide standardised SUV data while maintaining increased lesion detectability.

In the reconstruction modes identified as suitable candidates for harmonisation, a relatively higher increase was found in the recoveries of smaller spheres. This would lead to more “flat” RC curves, making subsequent quantitative analysis less dependent on lesion size. With the proposed reconstruction modes, the recoveries remained largely size-independent for ≥17 mm diameter lesions. Moreover, it is important to notice that a possible new harmonising standard for systems with PSF implies SUVmax recoveries to exceed 1.0. This suggests that if SUVmax remains the de facto field standard for PET/CT quantification, one should accept a positive bias of about 10 to 25% for larger homogeneous objects (≥17 mm diameter).

For both SUVmean and SUVmax the proposed reconstruction modes for harmonisation yielded promising results. The two largest spheres (28 mm diameter, 37 mm diameter) showed excellent agreement across all systems for both SUVmean and SUVmax. Even though there is not enough data for a reproducibility assessment, it can be predicted that a harmonising performance bandwidth is feasible for the next generation of PET/CT systems. The results from prospective validation using additional reconstructions will be further improved in the EARL accreditation process, where the centres will be guided to optimise their reconstruction settings in order to meet the new specifications.

As the harmonising RCs for SUVmean, SUVmax and SUVpeak all demonstrated a noticeable curve, the curvature and absolute error parameters exhibited increased or similar values with the initial reconstruction modes. Calculations excluding the smallest sphere demonstrated much better performance, which illustrated the high impact the smallest sphere has, that led to a significant decrease in the RCs range.

The utility of the SUVpeak was investigated as being a possible metric for standardised quantification. A recent prospective repeatability study by Kramer et al. [55] demonstrated the robustness of using the SUVpeak in non–small cell lung cancer patients. As previously shown by Makris et al. [56], and presented in supplemental Figs. 4–6, SUVpeak is significantly less sensitive to changes in reconstruction parameters and acquisition durations than SUVmean or SUVmax. The difference is mostly prominent in the initial group of 15 relatively loosely selected reconstruction modes, while within the five reconstructions for harmonisation and 23 additional ones, the difference became less apparent. On the other hand, the benefits of SUVpeak were offset by its consistently low recoveries for spheres with ≤17 mm diameter and therefore low MCR, which is comparable to that of SUVmean but significantly (20–40%) lower than that of SUVmax. This is due to peak VOI size approaching or even exceeding the size of the sphere, therefore missing some of the active volume. If this issue could be addressed by, for example, reducing the SUVpeak VOI size, SUVpeak may be become an effective alternative to SUVmax, especially if quantitative comparison among reconstructions of unknown origin or non-harmonised PET/CT systems is desired. Harmonisation among systems remains necessary in order to enable reliable use of SUVmax. Further studies are needed in order to explore the optimal peak VOI diameter maintaining noise cancelling effects, while producing higher, yet harmonised recoveries.

An alternative to the described methodology of achieving harmonised recoveries, such as suggested in this paper, could be to gradually increase the post smoothing on high recovery PET data until harmonised RC-s are obtained (supplemental Figs. 9–11). Such a method is available on some systems and previously validated by Lasnon et al. [54]. Potentially a post-smoothing feature on a workstation could be used for this purpose. This could result in higher recoveries and may reduce noise and Gibbs artefacts to acceptable levels for multicentre harmonisation. However, when offline post-smoothing needs to be applied to a dataset in order for it to achieve quantitative harmonisation, the filter information for the specific system always needs to accompany the PET data and extra care be taken that the filter be actually applied and clearly reported every time when required.

### Limitations and future directions

Quantification of PET images is affected by uncertainties derived from reconstruction settings as well as global system (cross-) calibration. In this study the experimental data were corrected for global calibration errors, but in clinical practice both effects should be taken into consideration. Therefore, an accurate system calibration remains of utmost importance for all PET/CT systems used for quantification in order to keep the uncertainties as low as possible.

The phantom experiments conducted were sensitive to measurement uncertainties of dose calibrators and human error during the phantom preparation phase. The uncertainties related to phantom filling procedure are not part of this study and may increase the bandwidth of achievable harmonisation.

All experiments on various PET/CT models were performed on appointed systems. The inter-system variability stemming from the individual differences among the systems of the same make and differences due to manufacturers allowed variability in well counter calibration factors, and may increase the bandwidth of achievable harmonisation even further, although the newly proposed harmonisation specification was set using the same bandwidth as current EARL, which was shown to be appropriate and feasible.

As the position of VOI-s used in the analysis and comparison of SUVmean data is based on PET images rather that CT data, it is to some extent affected by image noise and may induce a small additional uncertainty to the results. This, however, is reflective of the clinically used method of VOI positioning. When this strategy is followed, it is therefore important to also put a threshold on acceptable noise levels (in this paper background noise should be lower than 15%). Yet, use of CT-based VOI definition could be of interest in order to mitigate the effects of noise on VOI definition and subsequently on the measurement of the recovery coefficients. Another alternative could be the use of SUVpeak rather than SUVmax as a starting point for VOI definition, as was applied in Frings et al. [57]. These strategies may be considered when developing future standards.

Current study investigated harmonisation of PET/CT systems using ^18^F tracer based FDG. The results cannot be directly transferred to system performance harmonisation involving other PET isotopes such as ^68^Ga or ^82^Rb which have a substantially longer positron range. System performance harmonisation with positron emitting isotopes other then^18^F requires further investigation.

In this feasibility study we primarily made use of reconstruction methods and parameter settings that were predefined or could be easily set by the user on commercially released systems. Where the software permitted, we applied additional reconstructions to include at least PSF and TOF, and also tried other reconstruction parameter settings which were expected to yield higher recoveries than the current EARL specification. Yet, in this study we did not extensively explore a wide range of reconstruction settings as, e.g., iterations, subsets, matrix sizes, etc., since our aim was to investigate clinically available protocols which are accessible for the users. Moreover, the investigated reconstruction modes had similar, but still different, voxel sizes as well as the number of iterations/subsets between various systems which complicates direct comparison. In conclusion, the harmonisation investigated in this study should be considered as a first feasibility test aiming at improving the current EARL specifications. Of course, a higher level of harmonisation would also be possible by considering more parameters, but then the question will be the feasibly in clinical practice. Further work is also needed to more extensively explore the impact of PSF reconstructions, voxel size and number of iterations/subsets on the variability of quantitative metrics of clinical datasets. Some reports have already been published showing that the repeatability and ICC of SUVmax, SUVpeak and SUVmean are at an acceptable level [58].

To conclude, despite possible limitations, we have studied the feasibility of the harmonising state of the art PET/CT system performances, and the results suggest that an update of the EARL current specification is feasible and achievable in practice.

## Conclusions

This study investigated the feasibility of harmonising performance for PET/CT systems equipped with the latest Time-of-Flight (ToF) and resolution modelling (PSF) technology. Also, new possible specifications with higher contrast recoveries were investigated using various metrics such as average, maximum and peak SUV. Harmonising state of the art PET/CT systems with ToF and PSF technologies was found to be feasible. The harmonisation of such systems would require an update to the current multicentre accreditation program of EARL in order to accommodate higher recoveries. SUVpeak could be used as an uptake metric being less sensitive to noise and variation in image quality resulting from different reconstruction settings. It could be considered as an alternative to SUVmax if lower recoveries are considered to be acceptable for lesions of 17 mm in diameter and smaller.

## Electronic supplementary material

ESM 1 Fig. 4 Variable sphere size (**a to i**) SUVmean recovery coefficients of Siemens, Philips and GE reconstructions plotted as a function of background noise (CoV). Reconstructions determined to be suitable for harmonisation are marked with triangles of the corresponding colour. Fig. 5 Variable sphere size (**a to i**) SUVmax recovery coefficients of Siemens, Philips and GE reconstructions plotted as a function of background noise (CoV). Reconstructions determined to be suitable for harmonisation are marked with triangles of the corresponding colour. Fig. 6 Variable sphere size (**a to i**) SUVpeak recovery coefficients of Siemens, Philips and GE reconstructions plotted as a function of background noise (CoV). Reconstructions determined to be suitable for harmonisation are marked with triangles of the corresponding colour. Fig. 7 Transversal slices from harmonising reconstructions **a** – Siemens 1 – D; **b** – Siemens 2 – A; **c** – Philips – B; **d** – GE – F and **e** – GE – E. Colour scale represents SUV values. Fig. 8 CoV_BG_ values for initial 15 reconstruction modes (**a**) and 5 proposed harmonising reconstruction modes (**b**). Fig. 9 RC curves derived from post-filtered Siemens 1 - A reconstruction using SUVmean (a), SUVmax (b) and SUVpeak (c) quantitative metrics along with proposed new EARL specifications. Fig. 10 RC curves derived from post-filtered GE - A reconstruction using SUVmean (a), SUVmax (b) and SUVpeak (c) quantitative metrics along with proposed new EARL specifications. Fig. 11 RC curves derived from post-filtered Philips - A reconstruction using SUVmean (a), SUVmax (b) and SUVpeak (c) quantitative metrics along with proposed new EARL specifications. (DOCX 623 kb)
