# Supplementary material for: Feasibility of state of the art PET/CT systems performance harmonisation
Source: Eur J Nucl Med Mol Imaging. 2018 Mar 2;45(8):1344–61. doi: 10.1007/s00259-018-3977-4 (PMC5993859; doi:10.1007/s00259-018-3977-4)
Supplement: Supplementary file 1 — Fig. 4 Variable sphere size (a to i) SUVmean recovery coefficients of Siemens, Philips and GE reconstructions plotted as a function of background noise (CoV). Reconstructions determined to be suitable for harmonisation are marked with triangles of the corresponding colour. Fig. 5 Variable sphere size (a to i) SUVmax recovery coefficients of Siemens, Philips and GE reconstructions plotted as a function of background noise (CoV). Reconstructions determined to be suitable for harmonisation are marked with triangles of the corresponding colour. Fig. 6 Variable sphere size (a to i) SUVpeak recovery coefficients of Siemens, Philips and GE reconstructions plotted as a function of background noise (CoV). Reconstructions determined to be suitable for harmonisation are marked with triangles of the corresponding colour. Fig. 7 Transversal slices from harmonising reconstructions a – Siemens 1 – D; b – Siemens 2 – A; c – Philips – B; d – GE – F and e – GE – E. Colour scale represents SUV values. Fig. 8 CoVBG values for initial 15 reconstruction modes (a) and 5 proposed harmonising reconstruction modes (b). Fig. 9 RC curves derived from post-filtered Siemens 1 - A reconstruction using SUVmean (a), SUVmax (b) and SUVpeak (c) quantitative metrics along with proposed new EARL specifications. Fig. 10 RC curves derived from post-filtered GE - A reconstruction using SUVmean (a), SUVmax (b) and SUVpeak (c) quantitative metrics along with proposed new EARL specifications. Fig. 11 RC curves derived from post-filtered Philips - A reconstruction using SUVmean (a), SUVmax (b) and SUVpeak (c) quantitative metrics along with proposed new EARL specifications. (DOCX 623 kb) [file 259_2018_3977_MOESM1_ESM.docx]

# Supplemental Material

|  | **Siemens mCT** | **Siemens mCT Flow** | **GE 710** | **Philips Ingenuity** |
| --- | --- | --- | --- | --- |
| **Sensitivity** | 10 kcps/MBq | 10.2 kcps/MBq | 7.5 kcps/MBq | 7.3 kcps/MBq |
| **Peak NECR** | 186 kcps @ 30.1 kBq/ml | 180 kcps @ 28 kBq/ml | 130 kcps @ 29.5 kBq/ml | 124 kcps @ 2.3 kBq/ml |
| **TOF resolution** | 540 ps | 540 ps | N/A | 540 ps |
| **Crystal material** | LSO | LSO | Lutetium based | LYSO |
| **Crystal size** | 4 x 4 x 20 mm^3^ | 4 x 4 x 20 mm^3^ | 4.2 x 6.3 x 25 mm^3^ | 4 x 4 x 22 mm^3^ |
| **axial FOV** | 22.1 cm | 22.1 cm | 15.7 cm | 18 cm |
| **Resolution transverse 1cm** | 4.4 mm | 4.3 mm | 4.9 mm | N/A |
| **Resolution axial 1cm** | 4.4 mm | 4.3 mm | 5.6 mm | N/A |
| **Resolution transverse radial 10cm** | 5.7 mm | 5.2 mm | N/A | N/A |
| **Resolution transverse tangential 10cm** | 4.9 mm | 4.7 mm | N/A | N/A |
| **Axial 10 cm** | 5.9 mm | 5.9 mm | 6.3 mm | N/A |

**Supplemental table 1.** Specifications of the four PET/CT systems used for the initial 15 reconstruction modes

| **Parameter** | **GE 710, IQ & MI systems with Q.Clear option** | **GE 710, IQ & MI systems without Q.Clear** | **Philips Ingenuity TF** | **Siemens Biograph mCT Flow** | **Siemens Biograph mCT** |
| --- | --- | --- | --- | --- | --- |
| Bed position duration (s) | 300 | 300 | 300 | 0.5 mm/s (bed motion speed) | 300 |
| Recon method | QCFX,  beta = 800 | VPFXS | BLOB-OS-TF | TrueX-TOF (ultraHD-PET) | TrueX-TOF (ultraHD-PET) |
| Iterations/subsets | N/A | 2/24 | 3/33 | 2 / 21 | 3 / 21 |
| Matrix size | 256x256 | 256x256 | 144x144 | 200x200 | 400x400 |
| Post filter | N/A | 5.0mm | N/A | Gaussian, 5.0mm | Gaussian, 5.0mm |
| Z-filter | Standard | Standard | N/A | N/A | N/A |
| PSF (Point Spread Function) | ON | ON | ON | ON | ON |
| ToF (Time of Flight) | ON* | ON | ON | ON | ON |
| Attenuation correction | ON, CT based | ON, CT based | ON, CT based | ON, CT based | ON, CT based |
| Scatter correction | ON | ON | ON | ON | ON |

* Not available on Discovery IQ
**Supplemental table 2.** Requested acquisition and reconstruction parameters during validation part with the 18 additional systems

**Fig. 4** Variable sphere size (**a to i**) SUVmean recovery coefficients of Siemens, Philips and GE reconstructions plotted as a function of background noise (CoV). Reconstructions determined to be suitable for harmonisation are marked with triangles of the corresponding colour.

**Fig. 5** Variable sphere size (**a to i**) SUVmax recovery coefficients of Siemens, Philips and GE reconstructions plotted as a function of background noise (CoV). Reconstructions determined to be suitable for harmonisation are marked with triangles of the corresponding colour.

**Fig. 6** Variable sphere size (**a to i**) SUVpeak recovery coefficients of Siemens, Philips and GE reconstructions plotted as a function of background noise (CoV). Reconstructions determined to be suitable for harmonisation are marked with triangles of the corresponding colour.


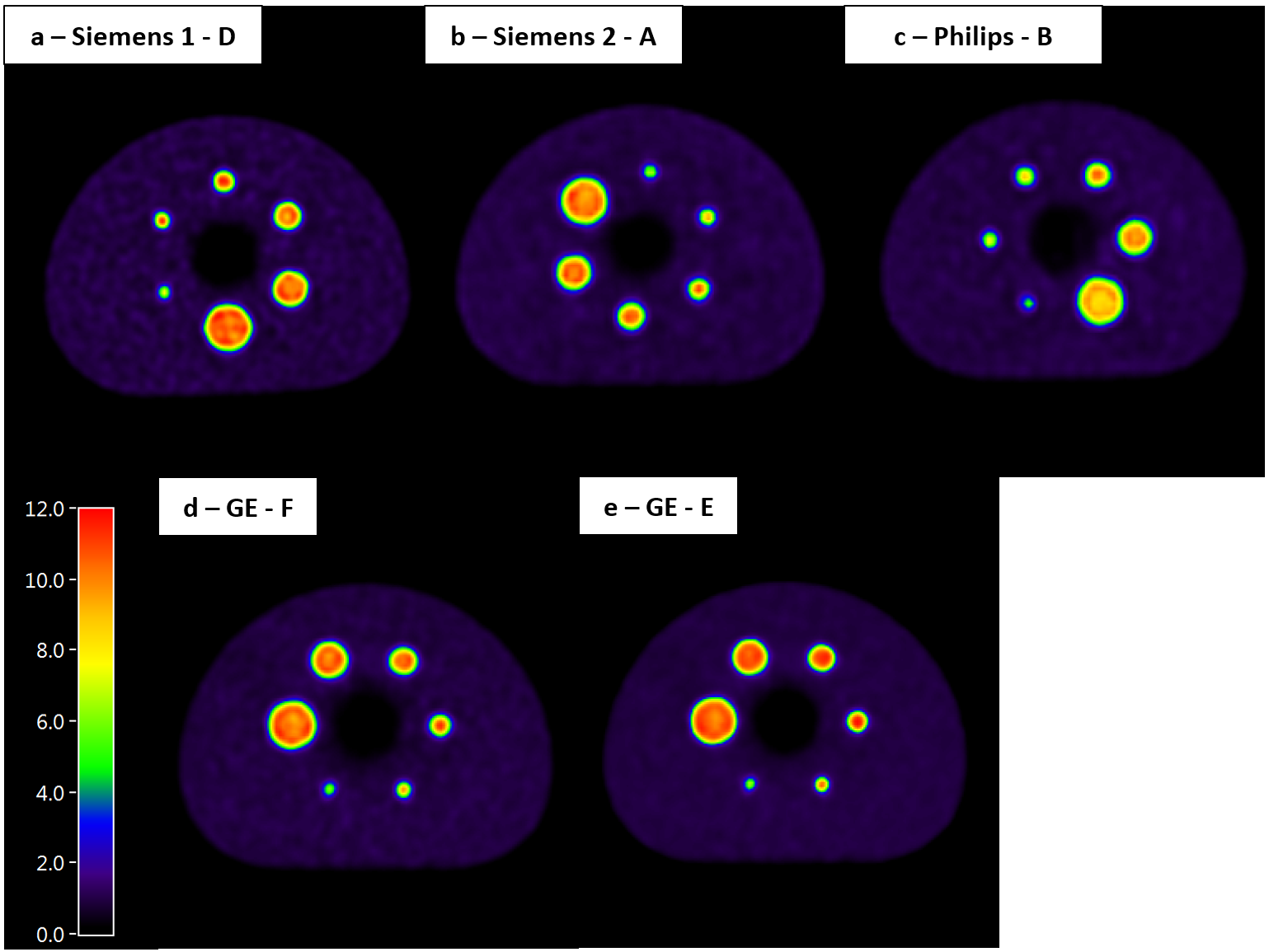


**Fig. 7** Transversal slices from harmonising reconstructions **a** – Siemens 1 – D; **b** – Siemens 2 – A; **c** – Philips – B; **d** – GE – F and **e** – GE – E. Colour scale represents SUV values.

**Fig. 8** CoV_BG_ values for initial 15 reconstruction modes (**a**) and 5 proposed harmonising reconstruction modes (**b**)

**Fig. 9** RC curves derived from post-filtered Siemens 1 - A reconstruction using SUVmean (a), SUVmax (b) and SUVpeak (c) quantitative metrics along with proposed new EARL specifications.

**Fig. 10** RC curves derived from post-filtered GE - A reconstruction using SUVmean (a), SUVmax (b) and SUVpeak (c) quantitative metrics along with proposed new EARL specifications.

**Fig. 11** RC curves derived from post-filtered Philips - A reconstruction using SUVmean (a), SUVmax (b) and SUVpeak (c) quantitative metrics along with proposed new EARL specifications.
